# Boswellic acids and malignant glioma: induction of apoptosis but no modulation of drug sensitivity

**DOI:** 10.1038/sj.bjc.6690419

**Published:** 1999-05

**Authors:** T Glaser, S Winter, P Groscurth, H Safayhi, E-R Sailer, H P T Ammon, M Schabet, M Weller

**Affiliations:** Laboratory of Molecular Neuro-Oncology, Department of Neurology, and Department of Pharmacology, Institute of Pharmaceutical Sciences, Hoppe-Seyler-Strasse 3, Tübingen, 72076, Germany; Department of Anatomy, University of Zürich, Zürich, Switzerland

**Keywords:** pentacyclic triterpenes, apoptosis, chemotherapy, brain tumour, glioma cells

## Abstract

Steroids are essential for the control of oedema in human malignant glioma patients but may interfere with the efficacy of chemotherapy. Boswellic acids are phytotherapeutic anti-inflammatory agents that may be alternative drugs to corticosteroids in the treatment of cerebral oedema. Here, we report that boswellic acids are cytotoxic to malignant glioma cells at low micromolar concentrations. In-situ DNA end labelling and electron microscopy reveal that boswellic acids induce apoptosis. Boswellic acid-induced apoptosis requires protein, but not RNA synthesis, and is neither associated with free radical formation nor blocked by free radical scavengers. The levels of BAX and BCL-2 proteins remain unaltered during boswellic acid-induced apoptosis. p21 expression is induced by boswellic acids via a p53-independent pathway. Ectopic expression of wild-type p53 also induces p21, and facilitates boswellic acid-induced apoptosis. However, targeted disruption of the p21 genes in colon carcinoma cells enhances rather than decreases boswellic acid toxicity. Ectopic expression of neither BCL-2 nor the caspase inhibitor, CRM-A, is protective. In contrast to steroids, subtoxic concentrations of boswellic acids do not interfere with cancer drug toxicity of glioma cells in acute cytotoxicity or clonogenic cell death assays. Also, in contrast to steroids, boswellic acids synergize with the cytotoxic cytokine, CD95 ligand, in inducing glioma cell apoptosis. This effect is probably mediated by inhibition of RNA synthesis and is not associated with changes of CD95 expression at the cell surface. Further studies in laboratory animals and in human patients are required to determine whether boswellic acids may be a useful adjunct to the medical management of human malignant glioma. © 1999 Cancer Research Campaign


*British Journal of Cancer* (1999) **80**(5/6), 756–765
© 1999 Cancer Research Campaign
Article no. bjoc.1998.0419

### Boswellic acids and malignant glioma: induction of apoptosis but no modulation of drug sensitivity

**T Glaser[1], S Winter[1], P Groscurth[3], H Safayhi[2], E-R Sailer[2], HPT Ammon[2], M Schabet[1] and M Weller[1]**

[1]Laboratory of Molecular Neuro-Oncology, Department of Neurology, and [2]Department of Pharmacology, Institute of Pharmaceutical Sciences, University of Tübingen, Hoppe-Seyler-Strasse 3, 72076 Tübingen, Germany; [3]Department of Anatomy, University of Zürich, Zürich, Switzerland

**Summary** Steroids are essential for the control of oedema in human malignant glioma patients but may interfere with the efficacy of chemotherapy. Boswellic acids are phytotherapeutic anti-inflammatory agents that may be alternative drugs to corticosteroids in the treatment of cerebral oedema. Here, we report that boswellic acids are cytotoxic to malignant glioma cells at low micromolar concentrations. In-situ DNA end labelling and electron microscopy reveal that boswellic acids induce apoptosis. Boswellic acid-induced apoptosis requires protein, but not RNA synthesis, and is neither associated with free radical formation nor blocked by free radical scavengers. The levels of BAX and BCL-2 proteins remain unaltered during boswellic acid-induced apoptosis. p21 expression is induced by boswellic acids via a p53-independent pathway. Ectopic expression of wild-type p53 also induces p21, and facilitates boswellic acid-induced apoptosis. However, targeted disruption of the p21 genes in colon carcinoma cells enhances rather than decreases boswellic acid toxicity. Ectopic expression of neither BCL-2 nor the caspase inhibitor, CRM-A, is protective. In contrast to steroids, subtoxic concentrations of boswellic acids do not interfere with cancer drug toxicity of glioma cells in acute cytotoxicity or clonogenic cell death assays. Also, in contrast to steroids, boswellic acids synergize with the cytotoxic cytokine, CD95 ligand, in inducing glioma cell apoptosis. This effect is probably mediated by inhibition of RNA synthesis and is not associated with changes of CD95 expression at the cell surface. Further studies in laboratory animals and in human patients are required to determine whether boswellic acids may be a useful adjunct to the medical management of human malignant glioma.

**Keywords:** pentacyclic triterpenes; apoptosis; chemotherapy; brain tumour; glioma cells

Cerebral oedema is a major cause of neurological disability and morbidity in human malignant glioma patients. Steroid medications are the most effective pharmacological agents for the control of cerebral oedema in human brain tumour patients. However, prolonged steroid therapy has significant side-effects, including mental changes, immunosuppression, Cushing syndrome and osteoporosis. Moreover, steroids stabilize blood–brain and blood–tumoural barriers, reduce tumour perfusion and inhibit apoptosis in human malignant glioma cells (Weller et al, 1997*a*; Naumann et al, 1998). These changes may interfere with the efficacy of adjuvant chemotherapy for malignant gliomas.

Alternative agents for the treatment of cerebral oedema in glioma patients are therefore urgently needed. Boswellic acids are phytotherapeutic agents obtained from the gum resin of *Boswellia serrata* which have been attributed anti-inflammatory properties and therapeutic effects, e.g. in inflammatory bowel disease and rheumatoid arthritis (Ammon, 1996; Gupta et al, 1997). These actions may be mediated by the inhibition of 5-lipoxygenase (5-LOX) (Safayhi et al, 1992, 1995; Sailer et al, 1996) and leucocyte elastase (Safayhi et al, 1997). Preliminary clinical data suggest that the drug formulation ‘H15’, which contains boswellic acids as putative active ingredients, has beneficial effects on the cerebral oedema in human glioma patients (Böker and Winking, 1997). Moreover, the same authors have reported that orally administered, tolerable doses of H15 (240 mg $kg^{-1}$) inhibit the growth of intracranially growing C6 gliomas in the rat (Winking et al, 1998).

*Received 19 June 1998*
*Revised 13 November 1998*
*Accepted 13 November 1998*

*Correspondence to:* M Weller

Here, we characterize the effects of boswellic acids on human glioma cells in-vitro and examine whether these agents interfere with apoptotic cell death of glioma cells after exposure to chemotherapeutic drugs and the CD95 ligand (CD95L).

### MATERIALS AND METHODS

### Chemicals

Amyrin, a mixture of isomeric α- and β-forms, was purchased from Roth (Karlsruhe, Germany). The boswellic acids, acetyl-11-keto-β-boswellic acid (AKBA), β-boswellic acid and acetyl-β-boswellic acid were isolated from the resin of *Boswellia serrata* and purified as described (Safayhi et al, 1992, 1995). All boswellic acids were dissolved in dimethylsulphoxide at 20 mM, except amyrin (4 mM). Aliquots were stored at –20°C. Cycloheximide (CHX), actinomycin D (ActD), doxorubicin, vincristine, cytarabine, N-tert-butyl-α-phenylnitrone (PBN), superoxide dismutase (SOD) and *N*-acetylcysteine (NAC) were purchased from Sigma (St Louis, MO, USA). Topotecan was obtained from SmithKline Beecham (King of Prussia, PA, USA), CCNU from Medac (Hamburg, Germany) and teniposide (VM-26) from Bristol (Munich, Germany). Radiochemicals were from Amersham (Braunschweig, Germany) and protease inhibitors from Bachem (Heidelberg, Germany). Terminal transferase, biotin-dUTP, streptavidin alkaline phosphatase conjugate, nitroblue tetrazolium chloride (NBT) and 5-bromo-4-chloro-3 indolyl phosphate (BCIP) for in situ DNA end labelling were obtained from Boehringer (Mannheim, Germany). 2′,7′-Dichlorodihydrofluorescein diacetate ($DCF-H_2$) was purchased from Molecular Probes (Groningen, The Netherlands). Soluble CD95L was obtained from CD95 ligand cDNA-transfected N2A neuroblastoma cells (Roth et al, 1997).

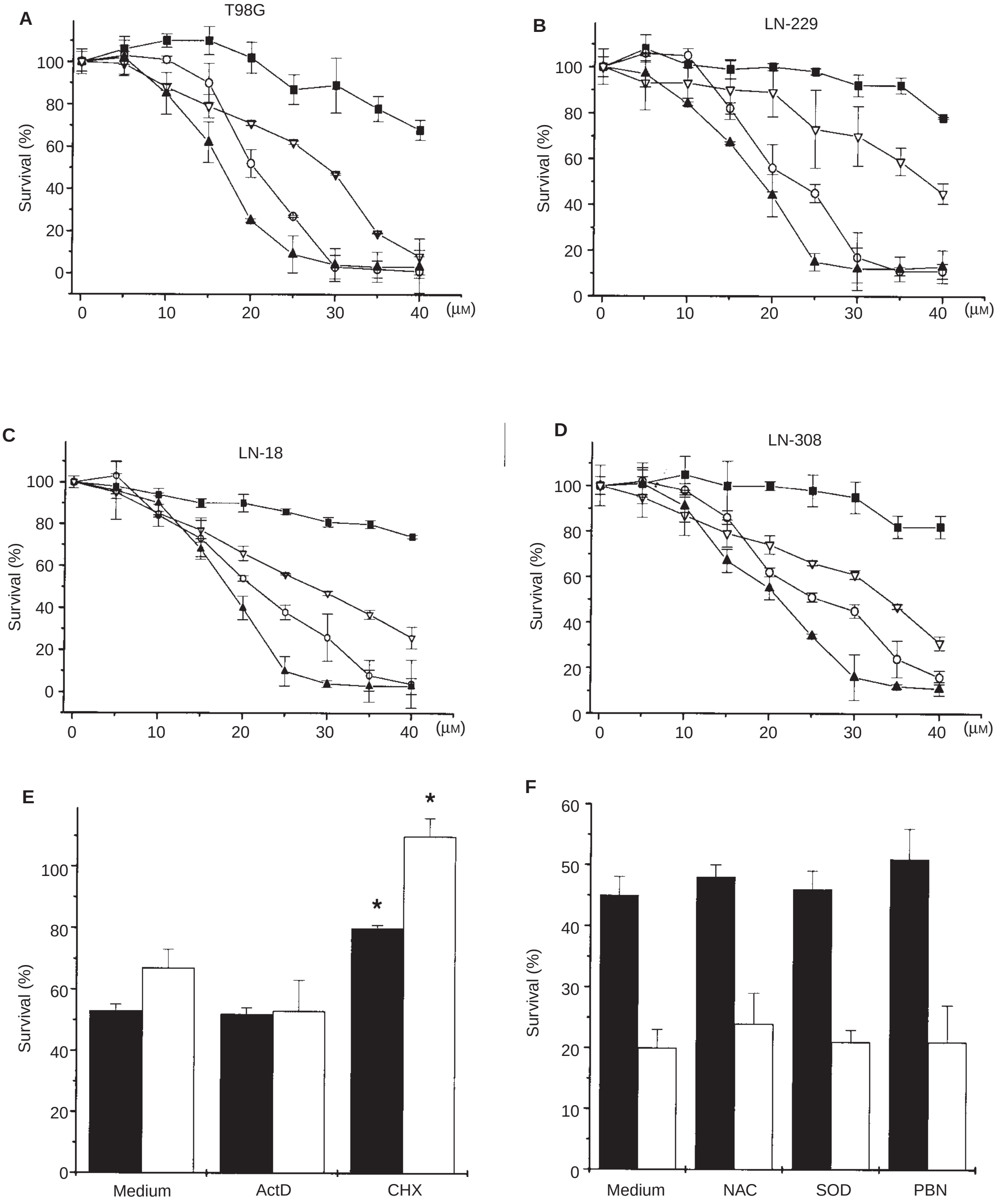

**Figure 1** Boswellic acid toxicity of human glioma cells: requirement for new protein synthesis but no role for reactive oxygen species. (**A–D**) T98G, LN-229, LN-18 or LN-308 human malignant glioma cells were seeded in 96-well plates ($10^4$ per well) for 24 h and then exposed to amyrin (■), AKBA (○), β-boswellic acid (▲) or acetyl-β-boswellic acid (△) for 48 h. Survival and proliferation were assessed by crystal violet assay. Data are expressed as mean percentages and s.e.m. ($n = 3$). (**E**) LN-229 (black bars) or T98G (open bars) cells were exposed to AKBA (20 μM) for 16 h in the absence or presence of ActD (0.5 μg $ml^{-1}$) or CHX (10 μg $ml^{-1}$). At these concentrations, RNA synthesis at 7–8 h was inhibited by ActD by 90% and 88%, and protein synthesis by CHX by 79% and 92% in LN-229 and T98G respectively (data not shown). Data are expressed as mean percentages of survival and s.e.m. relative to untreated cells or cells exposed to the macromolecular inhibitors alone (*$P < 0.05$, *t*-test). (**F**) LN-229 (black bars) or T98G (open bars) cells were treated with AKBA (20 μM) without or with a 2 h preexposure and a 48 h co-exposure to NAC (1 mM), SOD (50 U $ml^{-1}$) or PBN (100 μM). Data are expressed as in (**E**)

### Cell lines and cell culture

LN-18, LN-229 and LN-308 cells were kindly provided by Dr N de Tribolet (Lausanne, Switzerland). T98G were obtained from ATCC (Rockville, MD, USA). LN-229 cells are heterozygous for p53 and develop p53 transcriptional activity whereas LN-18 and T98G cells are mutant for p53. LN-308 cells do not express any p53 protein (Van Meir et al, 1994; Weller et al, 1998). The human glioma cell lines were cultured in Dulbecco's modified Eagle's medium (DMEM) supplemented with 10% fetal calf serum (FCS), 1 mM glutamine and 1% penicillin–streptomycin. HCT116 cells were cultured in McCoy's 5A medium supplemented with 10%

© Cancer Research Campaign 1999

FCS and 1% penicillin–streptomycin. Rat neonatal astrocytes were prepared as previously described (Weller et al, 1997*b*).

Cytotoxicity assays were performed in medium containing 5% FCS. The generation of glioma cell clones expressing murine BCL-2 or murine p53 val[135] proteins has been described (Weller et al, 1995*a*; Wagenknecht et al, 1997*b*; Trepel et al, 1998). Glioma cells expressing CRM-A were obtained by electroporation (Biorad Gene Pulser, 250 V, 950 μF) using the pEFR CRM-A flag puro plasmid (Strasser et al, 1995). Transgene expression was assessed by immunoblot analysis for murine BCL-2 and p53 protein (Weller et al, 1995*a*; Trepel et al, 1998) and by Northern blot analysis for CRM-A mRNA (Wagenknecht et al, 1998). Experiments were performed with control transfectants harbouring plasmids without insert (neo for BCL-2, hygro for p53, puro for CRM-A).

### Viability and apoptosis studies

The procedures for crystal violet staining, in-situ DNA end labelling, quantitative DNA fluorometry and electron microscopy have been published in detail (Weller et al, 1994, 1997*b*). The MTT assay (3-(4,5-dimethylthiazal-2-yl)-2,5-diphenyltetrazolium bromide) was used to determine viability in HCT116 colon carcinoma cells.

### Biochemical studies

For the determination of RNA synthesis, the cells were grown in 12-well plates ($10^4$ cells $ml^{-1}$) and treated as indicated for 8 h. The cells were pulse labelled with 0.5 μCi $ml^{-1}$ [5,6-$^3$H]uridine (specific activity: 40 Ci $mmol^{-1}$) from 7–8 h, washed with ice-cold phosphate-buffered saline (PBS) (2×) and ice-cold 6% trichloroacetic acid (2×) to remove unincorporated, acid-soluble label, and lysed with 0.1 N NaOH (1 ml) overnight at room temperature. Then, 0.5 ml of the lysate was mixed with 4 ml scintillation cocktail and counted in a liquid scintillation counter. For the determination of protein synthesis, the cells were pulse labelled during the last hour of incubation with 1 μCi $ml^{-1}$ L-[4,5-$^3$H]leucine (specific activity: 167 Ci $mmol^{-1}$). After washing with ice-cold PBS (3×), the cells were lysed with 0.1% sodium dodecyl sulphate (SDS) (0.5 ml per well) for 30 min at 37°C. Proteins were precipitated by addition of ice-cold 15% trichloroacetic acid (0.5 ml per well) and pelleted by centrifugation (13 000 rpm, 10 min, 4°C). The supernatant (trichloroacetic acid-soluble fraction) was counted in a liquid scintillation counter after addition of 5-ml scintillation cocktail. The pellet (trichloroacetic acid-precipitable fraction) was washed with 6% trichloroacetic acid (3×), dissolved in 0.5 ml 0.1 N NaOH, and the radioactivity of the precipitated proteins measured after addition of 5-ml scintillation cocktail.

Cleavable DNA topoisomerase complex formation induced by classical inhibitors of topoisomerase I or II was assessed by potassium chloride precipitation as described (Rowe et al, 1986; Winter et al, 1998).

The generation of reactive oxygen species was monitored using the fluorescent probe, DCF-$H_2$ (Reber et al, 1998). Immunoblot analysis was performed as described (Weller et al, 1994). The primary antibodies were anti-p53 Ab-2 (Oncogene Science, Uniondale, NY, USA; 2.5 μg $ml^{-1}$), anti-BCL-2 (Dakopatts, Glostrup, Denmark; 2 μg $ml^{-1}$) and anti-BAX (Santa Cruz, CA, USA; 2 μg $ml^{-1}$). Flow cytometric analysis of CD95 expression was performed as described (Weller et al, 1995*b*) using fluorescein isothiocyanate (FITC)-UB2 anti-Fas antibody from Immunotech (Hamburg, Germany) and FITC-mouse $IgG_1$ from Sigma (St Louis,

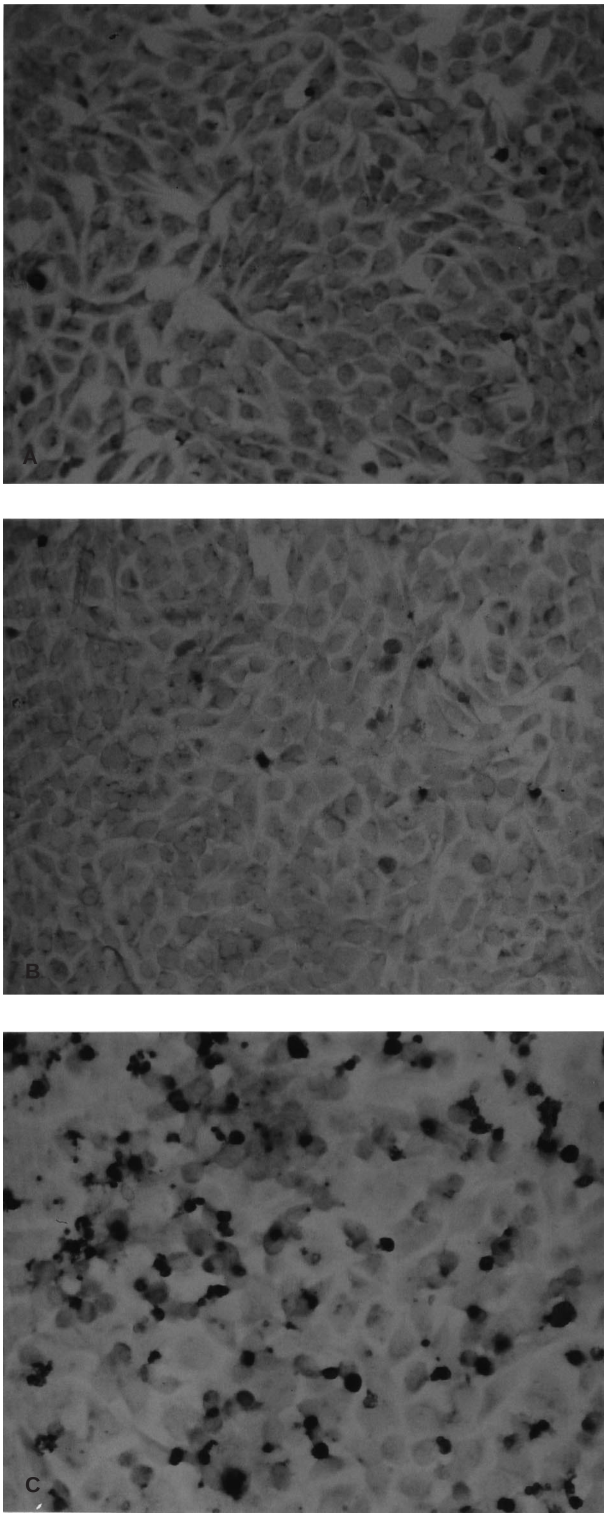

**Figure 2** Induction of apoptosis in human LN-229 malignant glioma cells by AKBA, but not amyrin. LN-229 cells were either untreated (**A**) (× 250) or exposed to 20 μM amyrin (**B**) (× 250) or 20 μM AKBA (**C**) (× 400) for 48 h and processed for in-situ DNA end labelling as described (Weller et al, 1994, 1997*b*) to detect the presence of DNA breaks characteristic of apoptosis. Note the high number of labelled nuclei after exposure to AKBA. No specific labelling was obtained when the co-factor for terminal transferase, cobalt chloride, was omitted

*© Cancer Research Campaign 1999*

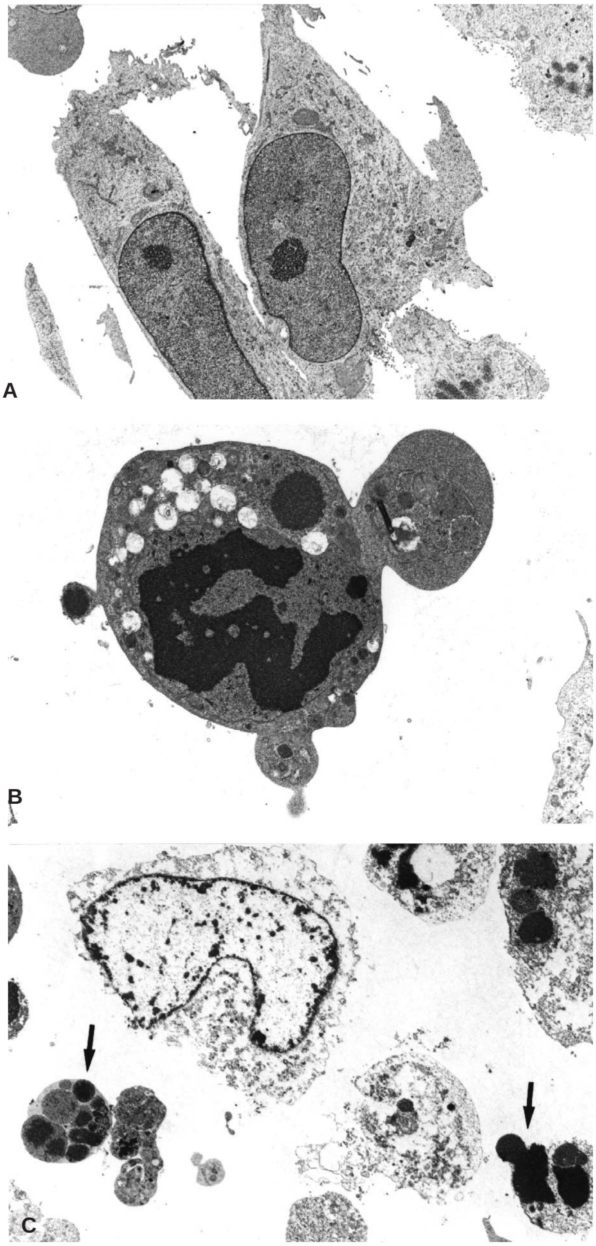

**Figure 3** AKBA-induced apoptosis of LN-229 cells: transmission electron microscopy. LN-229 cells were exposed to AKBA (20 μM) for 24 h or 48 h and processed for electron microscopy as described (Weller et al, 1994). (**A**) (× 2500) shows the appearance of untreated cells, (**B**) and (**C**) show different stages of apoptosis 24 h and 48 h after treatment with AKBA. In (**B**) (× 5700), there is a typical early apoptosis with cytoplasmic blebbing and condensed nuclear heterochromatin. In (**C**) (× 2200), both apoptotic (arrows) and necrotic cells are visible

MO, USA) as isotype control antibody. Immunolabelling was quantified as specific fluorescence index (SFI) defined by the ratio of the mean cellular fluorescence with specific versus control antibody.

### Cell cycle analysis

For cell cycle analysis, the glioma cells were treated as indicated, washed with PBS, incubated with trypsin for 3 min at 37°C, harvested, washed and fixed with 70% ice-cold ethanol. A total of $10^6$ cells per condition were stained with propidium iodide (50 μg $ml^{-1}$) in PBS containing 100 U $ml^{-1}$ RNase A) for 30 min. All samples were analysed on an FacsCalibur flow cytometer using the Cell Quest acquisition and analysis software (Becton Dickinson, Heidelberg, Germany).

### RESULTS

### Boswellic acids induce apoptosis in human malignant glioma cells

To determine cytotoxic and antiproliferative effects of the boswellic acids, the glioma cells were exposed to different boswellic acids for 0–48 h. Figure 1A–D shows that all four glioma cell lines were susceptible to boswellic acid-induced growth inhibition in a concentration-dependent manner and that the order of potency of the compounds evaluated was similar in all cell lines. Acetyl-β-boswellic acid was most potent, followed by AKBA and β-boswellic acid, whereas amyrin had only minor effects. When the cells were seeded at low density, exposed to the drugs for 24 h, and then allowed to recover for 5–10 generation times, there was no significant decrease in the $EC_{50}$ values (data not shown), suggesting that the acute cytotoxic effects shown in Figure 1A–D are responsible for the inhibition of clonogenic survival in the pulse/recovery colony formation assays. Next, we asked whether the susceptibility to boswellic acids was specific for the neoplastic phenotype of glioma cells or could also be observed in untransformed rat neonatal astrocytes. To this end, we exposed the astrocytes to the boswellic acids as done with the glioma cells in Figure 1. We found that the proliferating neonatal rat astrocytes were no more resistant to boswellic acid cytotoxicity than the malignant glioma cell lines, that is, approximate $EC_{50}$ values for cytotoxicity are 20 μM for AKBA, 27 μM for acetyl-β-boswellic acid and 40 μM for β-boswellic acid.

Light microscopic monitoring suggested that boswellic acid-induced growth inhibition of glioma cells was associated with the induction of apoptosis. This was confirmed by in situ DNA end labelling (Figure 2) and electron microscopy (Figure 3). In-situ DNA end labelling revealed a high number of cells harbouring DNA breaks after exposure to AKBA, whereas there was no difference in labelling between untreated cells and amyrin-treated cells (Figure 2), corresponding to the toxicity data (Figure 1 A–D). Similarly, AKBA, but not amyrin, induced classical features of apoptotic cell death as assessed by electron microscopy (Figure 3). Exposure to AKBA, but not amyrin, induced a moderate degree of DNA fragmentation up to 10% of the total DNA, a value that is consistent with the minor degree of DNA fragmentation induced by other pro-apoptotic stimuli in these cells, including CD95L (data not shown).

### AKBA-induced glioma cell apoptosis requires new protein synthesis but does not involve reactive oxygen species formation

AKBA-induced cell death was attenuated by a protein synthesis inhibitor, CHX, but unaffected by the RNA synthesis inhibitor, ActD (Figure 1E), suggesting that translation of preexisting mRNA mediates AKBA toxicity. AKBA did not induce the formation of reactive oxygen species (data not shown), and the antioxidants NAC, SOD and PBN did not block AKBA toxicity (Figure 1F). Ethacrynic acid was used as a positive control for reactive oxygen species formation (Reber et al, 1998), and

© Cancer Research Campaign 1999

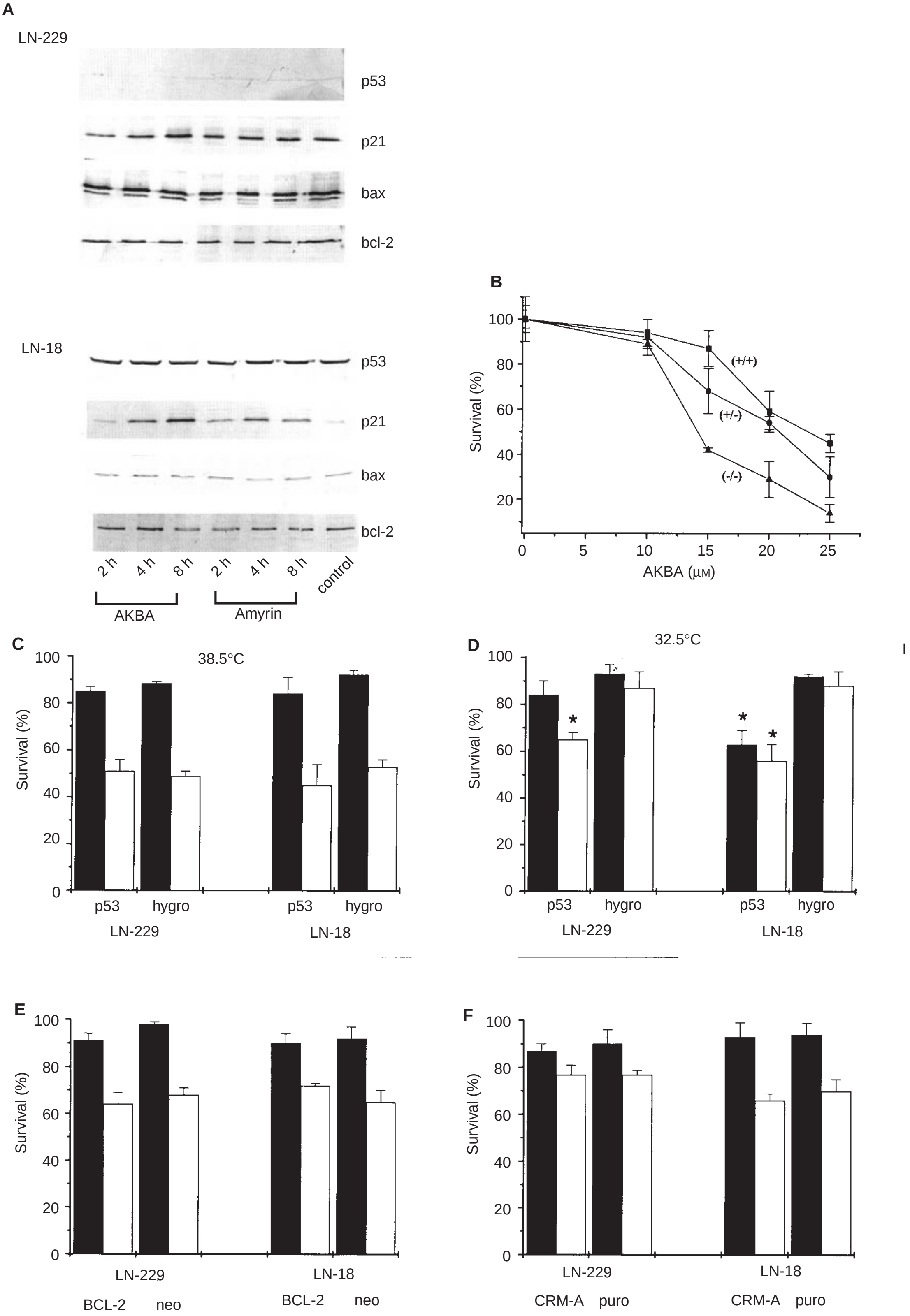

**Figure 4** Boswellic acid toxicity: the role of apoptosis control gene products. (**A**) LN-229 cells (upper panel) and LN-18 (lower panel) were either untreated (control) or exposed to AKBA or amyrin at 20 μM. After 2, 4 or 8 h p53, BAX, BCL-2 and p21 protein expression were examined by immunoblot analysis as described (Weller et al, 1997*c*). (**B**) Parental HCT116 human colon carcinoma cells (p21 +/+, ■) or sublines with heterozygous (+/–, •) or homozygous (–/–, △) p21 gene deletions were seeded in 96-well plates ($10^4$ per well) for 24 h and then exposed to AKBA for 48 h. Survival was assayed by the MTT assay. Data are mean percentages and s.e.m. ($n$ = 3). (**C**, **D**) LN-229 and LN-18 hygro or p53-transfected cells were maintained at 38.5°C and treated with AKBA (10 μM, black bars; 20 μM, open bars) for 48 h (**C**) or shifted to 32.5°C for 4 h prior to AKBA exposure for 48 h (**D**) (30 μM, black bars; 40 μM, open bars) (*$P$ < 0.05, $t$-test). (**E, F**) LN-229 and LN-18 neo or BCL-2-transfected cells (**E**), or LN-229 and LN-18 puro or CRM-A-transfected cells (**F**) were exposed to AKBA at 10 μM (black bars) or 20 μM (open bars). Data in **B–F** are expressed as mean percentages of survival and s.e.m. ($n$ = 3)

 *© Cancer Research Campaign 1999*

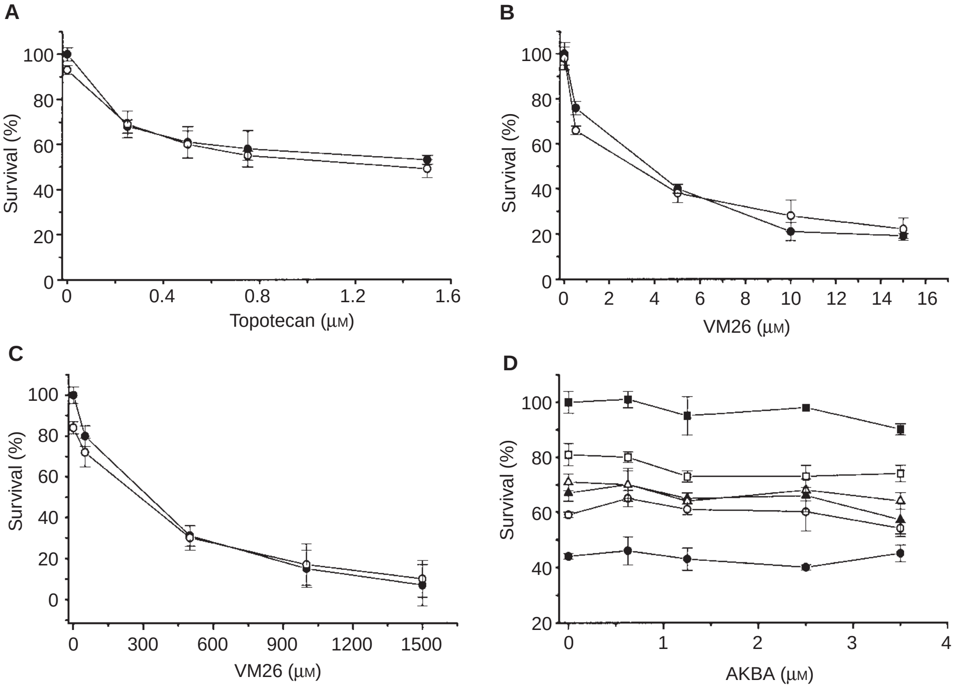

**Figure 5** AKBA does not modulate cancer drug toxicity of human malignant glioma cells. LN-229 cells were either untreated or pretreated with 2.5 μM AKBA for 24 h and then exposed to topotecan (**A**), VM-26 (**B**) or CCNU (**C**) in the absence (filled symbols) or presence (open symbols) of AKBA (2.5 μM). (**D**) LN-229 cells were untreated or pretreated to increasing concentrations of AKBA for 24 h and then co-exposed to the drugs for 72 h (■, AKBA only; □, 0.25 μM doxorubicin; ●, 4 μM VM26; ○, 50 μM cytarabine; ▲, 0.5 μM topotecan; △, 150 μM CCNU). Survival was assessed by crystal violet assay. Data are expressed as mean percentages of survival and s.e.m. (*n* = 3)

potassium-deprived cerebellar granule neurons served as a biological control for the prevention of apoptosis by the antioxidants (Schulz et al, 1996).

### Boswellic acid toxicity: the role of p53, p21, BCL-2, BAX and caspases

To examine the role of major apoptosis control genes in boswellic acid-induced apoptosis, we exposed the glioma cells to AKBA and monitored the expression of p53, p21, BCL-2 and BAX proteins. Figure 4A shows that AKBA neither induces p53 protein accumulation in LN-229, which are wild-type for p53, nor in LN-18, which express mutant p53 protein. AKBA also failed to alter the levels of BAX or BCL-2 proteins, except for a minor reduction of BCL-2 protein at 8 h after AKBA exposure in LN-18 cells. Interestingly, there was a time-dependent accumulation of p21 in both cell lines after exposure to AKBA (20 μM), including p53-mutant LN-18 cells, indicating that p21 expression is induced in a p53-independent manner.

p21 inhibits cyclin-dependent kinase (cdk) activity and mediates the p53-controlled cell cycle arrest in G0/1. However, cell cycle analysis after exposure to AKBA revealed no specific pattern of cell cycle redistribution in LN-229 or LN-18 cells despite the induction of p21 expression (data not shown). This may be a consequence of the RB checkpoint disruption in both cell lines which we have been able to link to a homozygous deletion of p16 in both cell lines (Wagenknecht et al, 1997*b*; Weller et al, 1998).

To further examine the role of p21 in AKBA toxicity, we compared the effects of AKBA toxicity in HCT116 colon carcinoma cells which were either wild-type or had a heterozygous or homozygous deletion of the p21 gene (Waldman et al, 1995, 1996). The wild-type cells expressed high levels of p21 constitutively that were not enhanced further by AKBA (data not shown). The wild-type cells were more resistant to AKBA than the sublines with heterozygous or homozygous p21 deletions (Figure 4B), suggesting that p21 is not a down-stream effector of AKBA toxicity but confers relative protection from apoptosis, as has been hypothesized for p53-mediated p21 induction (Gomez-Manzano et al, 1997; Trepel et al, 1998).

To examine the role of p53 in AKBA toxicity in more detail, we took advantage of LN-18 and LN-229 transfectants expressing the temperature-sensitive p53 val$^{135}$ mutant that behaves as a mutant p53 protein at 38.5°C but adopts wild-type conformation and activity at 32.5°C (Michalowitz et al, 1990; Trepel et al, 1998). At 38.5°C (mutant p53), the p53-transfected cells were as sensitive to AKBA as hygro control cells (Figure 4C). The shift from 38.5°C to 32.5°C per se reduced drug sensitivity, presumably due to significantly slower proliferation of hygro control cells, reduced metabolic activity and growth arrest of p53 clones at this temperature (Wagenknecht et al, 1997*b*). Compared with isogenic hygro controls, wild-type p53-expressing cells (32.5°C) were more sensitive to AKBA-induced apoptosis (Figure 4D), even though p21 is induced by p53 under these conditions (Trepel et al, 1998).

Next, we examined whether ectopic expression of BCL-2 or of the viral caspase inhibitor, CRM-A, affected AKBA toxicity. Figure 4E and F show that neither BCL-2 nor CRMA-A protected LN-18 or LN-229 cells from AKBA-induced apoptosis. To confirm that caspases were not involved in AKBA toxicity of glioma cells, we showed that the caspase 1 (ICE)-type peptide

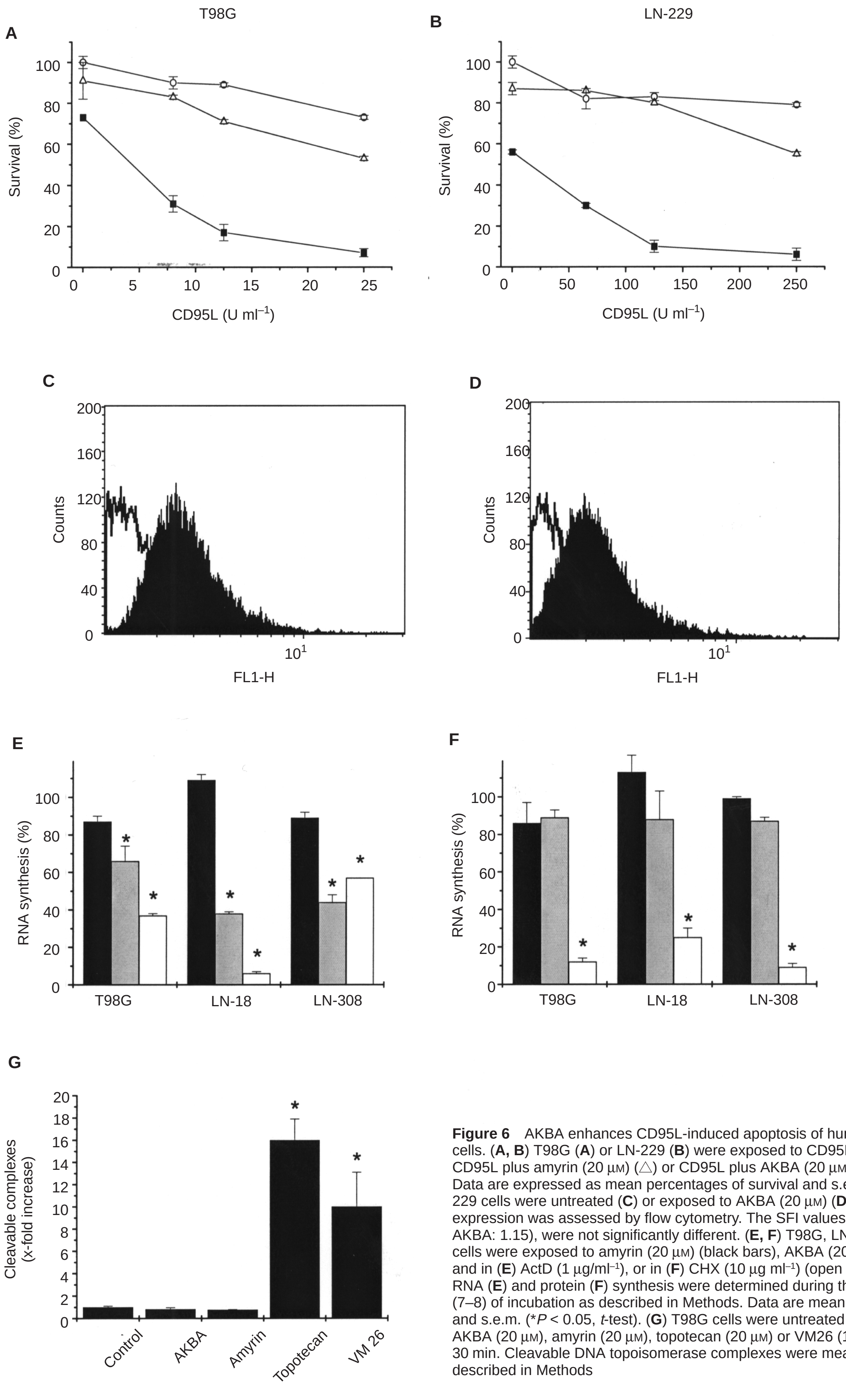

**Figure 6** AKBA enhances CD95L-induced apoptosis of human glioma cells. (**A, B**) T98G (**A**) or LN-229 (**B**) were exposed to CD95L alone (○) or CD95L plus amyrin (20 µM) (△) or CD95L plus AKBA (20 µM) (■) for 48 h. Data are expressed as mean percentages of survival and s.e.m. (**C, D**) LN-229 cells were untreated (**C**) or exposed to AKBA (20 µM) (**D**) for 24 h. CD95 expression was assessed by flow cytometry. The SFI values (control: 1.35; AKBA: 1.15), were not significantly different. (**E, F**) T98G, LN-18 or LN-308 cells were exposed to amyrin (20 µM) (black bars), AKBA (20 µM) (grey bars) and in (**E**) ActD (1 µg/ml$^{-1}$), or in (**F**) CHX (10 µg ml$^{-1}$) (open bars) for 8 h. RNA (**E**) and protein (**F**) synthesis were determined during the last hour (7–8) of incubation as described in Methods. Data are mean percentages and s.e.m. (*$P < 0.05$, *t*-test). (**G**) T98G cells were untreated or exposed to AKBA (20 µM), amyrin (20 µM), topotecan (20 µM) or VM26 (10 µM) for 30 min. Cleavable DNA topoisomerase complexes were measured as described in Methods

 © Cancer Research Campaign 1999

**Table 1** Summary of data on boswellic acid cytotoxicity of human malignant glioma cells

| | **Role in boswellic acid-induced apoptosis of human glioma cells** |
|---|---|
| CD95 | No changes in CD95 expression at the cell surface |
| CD95L | Synergy with boswellic acids in inducing apoptosis |
| Bax | Expression levels remain unaltered |
| Bcl-2 | Expression levels remain unaltered |
| p53 | No activation, but wild-type p53 gene transfer facilitates boswellic acid-induced apoptosis |
| p21 | Induced, appears to be implicated in protection from boswellic acid-induced apoptosis |
| Caspases | No role for CRM-A-, YVAD-or DEVD-sensitive caspases |

inhibitor, Ac-YVAD-chloromethylketone (YVAD-CMK), and the caspase 3 (CPP32)-type peptide inhibitor, aspartic acid aldehyde (DEVD-CHO), failed to inhibit AKBA toxicity (data not shown). In contrast, ectopic expression of BCL-2 or CRM-A or exposure to the peptide inhibitors attenuated CD95 ligand-induced apoptosis of these cells (Weller et al, 1995*a*) (data not shown).

### Boswellic acids do not modulate chemotherapeutic drug-induced cytotoxicity but synergistically enhance CD95L-induced apoptosis via inhibition of RNA synthesis

In contrast to dexamethasone, which inhibits chemotherapeutic drug-induced apoptosis of glioma cells (Weller et al, 1997*a*), subtoxic concentrations of AKBA did not interfere with the cytotoxic and anticlonogenic actions of several chemotherapeutic drugs, including cytarabine, CCNU, doxorubicin, topotecan, vincristine and VM26. There was also no augmentation of drug toxicity by AKBA. These experiments were done with T98G and LN-229 cells and were performed as in Figure 1A for acute cytotoxicity, and using a 24 h pulse at very low seeding density with 5–10 generation times recovery for assessing effects on clonogenicity. We evaluated combinations of three concentrations of AKBA and four concentrations of each cancer chemotherapy drug. A representative example of these overall negative data is illustrated in Figure 5.

Conversely, CD95L-induced apoptosis was synergistically enhanced by AKBA (Figure 6 A,B). This effect was not related to AKBA-induced increases in CD95 expression (Figure 6 C,D). Potentiation of apoptosis by inhibitors of RNA and protein synthesis such as ActD or CHX is paradigmatic for the cell death induced by cytokines such as CD95L and tumour necrosis factor (TNF)-α (Weller et al, 1994). Similarly, the concentrations of AKBA required for synergistic glioma cell killing (Figure 6A) induced prominent inhibition of RNA synthesis (Figure 6E), suggesting that an ActD-like effect mediates synergy of AKBA with CD95L. Protein synthesis was not affected (Figure 6F).ActD and CHX served as positive controls for the inhibition of RNA and protein synthesis. Parallel experiments assessing the concentration–response relation for the effects of AKBA on (i) CD95L-induced glioma cell killing and (ii) inhibition of RNA synthesis revealed that both effects were intimately linked (data not shown). In contrast to classical inhibitors of topoisomerase I or II, e.g. camptothecin or VM26, which were used as positive controls, AKBA did not induce the formation of cleavable DNA topoisomerase complexes (Figure 6G).

### DISCUSSION

Boswellic acids are putative 5-LOX inhibitors which may have a role in the management of various inflammatory conditions and in the control of cerebral oedema in brain tumour patients. These agents seem to block 5-LOX activity by direct interaction with the enzyme at a binding site different from the catalytic centre. Recent data support that the planar pentacyclic ring system is crucial for enzyme binding, but only compounds containing a hydrophilic group on C-4 and a keto-function on C-11, e.g. AKBA, are able to inhibit 5-LOX activity completely. Modulation or elimination of these two functional groups attenuates (e.g., β-boswellic acid and acetyl-β-boswellic acid) or abrogates (e.g. amyrin) the inhibitory effect on 5-LOX activity. Here, we show that boswellic acids induce apoptosis in human malignant glioma cells (Figures 1–3, Table 1). While acetyl-β-boswellic acid is the most toxic substance among the tested boswellic acids, AKBA is the most potent 5-LOX inhibitor, with an $IC_{50}$ of 1.5 μM in $Ca^{2+}$/ionophore-stimulated intact polymorphonuclear neutrophils (Safayhi et al, 1992) and presumably in glioma cells (Heldt et al, 1997). Importantly, amyrin, which is devoid of toxic effects, is also not a LOX inhibitor, suggesting that the functional groups responsible for inhibition of 5-LOX activity also mediate the effects on viability of the glioma cells. Yet, the $IC_{50}$ values for LOX inhibition are 10 to 20-fold lower than those required for cytotoxicity (Figure 1 A–D) (Heldt et al, 1997).

We find that AKBA-induced apoptosis requires new protein, but not RNA synthesis, and does not involve the generation of reactive oxygen species (Figure 1 E,F). The levels of BAX and BCL-2 are unaltered, and ectopic expression of BCL-2 does not inhibit apoptosis (Figure 4 A,E). The failure of ectopic CRM-A expression or pseudosubstrate enzyme inhibitors to abrogate apoptosis indicates that caspase activation may not be required for AKBA-induced apoptosis (Figure 4F). These data make sense in that caspase-dependent apoptotic cell deaths are predicted to be inhibited by BCL-2, as e.g. CD95-mediated apoptosis in glioma cells (Wagenknecht et al, 1998).

Exposure to cytotoxic concentrations of AKBA does not activate p53 activity in a wild-type p53-containing cell line, LN-229 (Figure 4A). However, ectopic expression of a temperature-sensitive p53 $val^{135}$ mutant facilitates AKBA-induced apoptosis when the grafted protein is expressed at wild-type conformation (Figure 4D). p21, the major p53 response protein associated with growth arrest in G0/1, was induced by AKBA in cells with wild-type (LN-229) or mutant (LN-18) p53 status (Figure 4A), suggesting that AKBA activates a p53-independent pathway of p21 expression.

© Cancer Research Campaign 1999 

Interestingly, AKBA-induced p21 expression did not result in a growth arrest in G0/1 or G2/M. However, when glioma cells are growth-arrested by p53 val$^{135}$ in wild-type conformation, p21 is also induced (Wagenknecht et al, 1997*b*) but the cells stably arrest in G2/M (Weller, unpublished observation), suggesting that p21 expression per se does not determine the type of cell cycle arrest in glioma cells. That p53-dependent p21 expression failed to induce G0/1 arrest in LN-18 and LN-229 cells, was consistent with the disruption of the RB checkpoint in these cells, a target of p21-mediated G0/1 arrest, via loss of both p16 alleles (Weller et al, 1998).

The facilitation of AKBA-induced apoptosis by wild-type p53 which also induces p21, as well as the p53-independent induction of p21 by AKBA, suggested a role for p21 as an effector of AKBA-induced apoptosis. To clarify this issue, we compared the isogenic wild-type human colorectal cancer cell line HCT116 with sublines with heterozygous or homozygous p21 gene disruptions for their sensitivity to AKBA (Figure 4B).

Interestingly, p21 disruption in these cells enhanced rather than decreased sensitivity to apoptosis. Thus, although these cells did not up-regulate p21 protein expression in response to AKBA and may not reflect the biology of glial tumour cell lines, these observations imply a cytoprotective rather than apoptosis messenger role for p21. Interestingly, p21 has been found to accumulate in gliomas in vivo (Jung et al, 1995) and may mediate the cytoprotective effects of steroids in glioma cells (Naumann et al, 1998). Further, p21 is now attributed a general role in inhibiting drug-induced apoptosis in glial and non-glial tumour cells (Sheikh et al, 1997; Ruan et al, 1998).

CD95 is a novel target of immunotherapy for malignant glioma (Weller et al, 1994; Weller et al, 1995*a*). Interestingly, the LOX inhibitor, NDGA, inhibits CD95L-induced apoptosis of glioma cells (Wagenknecht et al, 1997*a*). Yet, boswellic acids that are also LOX inhibitors synergize with CD95L in inducing apoptosis (Figure 6). Thus, NDGA may block apoptosis via mechanisms unrelated to LOX inhibition or boswellic acids synergistically enhance CD95L-induced apoptosis independent of LOX inhibition. Here, the concentrations required for boswellic acid-mediated augmentation of CD95L-induced apoptosis probably exceed those required for LOX inhibition (Sailer et al, 1996; Heldt et al, 1997), but parallel those required for inhibition of RNA synthesis (Figure 6E), suggesting that an ActD-like effect (Weller et al, 1994) is responsible for the synergy of AKBA and CD95L. Thus, AKBA is a rather unique pro-apoptotic agent that inhibits RNA synthesis (Figure 6E) but requires new protein synthesis, possibly from mRNAs with a long half-life, to mediate toxicity (Figure 1E). While preliminary data indicate that boswellic acids inhibit topoisomerase I and II activity (Heldt et al, 1997; Hoernlein et al, 1997), this effect of boswellic acids differs from those of classical inhibitors, such as camptothecin or teniposide, which induce cleavable DNA/topoisomerase I/II complex formation in the glioma cells (Weller et al, 1997*c*) (Figure 6G). Incidently, we find that potentiation of CD95L-induced apoptosis of human glioma cells by camptothecin and topotecan, similar to that of boswellic acids (Figure 6E), correlates with inhibition of RNA synthesis (Winter and Weller, 1998). Boswellic acid-induced changes of CD95 expression were excluded as a mechanism of synergy (Figure 6 C,D). Note that the same concentrations of AKBA that sensitize for acute CD95L-induced apoptosis kill glioma cells upon longer exposure by an apoptotic pathway that requires new protein synthesis (Figure 1E).

At present, defined drug formulations containing boswellic acids are not available, and the preliminary putatively beneficial effects of boswellic acids on the oedema of glioma patients (Böker and Winking, 1997) were obtained with tablets that contain undetermined amounts of various boswellic acids. Thus, one may not judge systemic toxicity and anti-oedema activity of pure boswellic acids. Yet, the low systemic toxicity of the available drug formulations (Gupta et al, 1997) is certainly promising. From a clinical point of view, the lack of interference of boswellic acids with chemotherapeutic drug-induced apoptosis of glioma cells (Figure 5) is highly relevant. We are currently performing a phase I/II clinical trial to confirm the efficacy of boswellic acids for oedema control in glioma patients with prominent cerebral oedema. Such patients currently depend on steroid medications which have significant side-effects and probably limit the efficacy of chemotherapy (Weller et al, 1997*a*). The anti-oedema actions of boswellic acids alone would make them a valuable addition to the limited medical options in the treatment of malignant glioma, specifically because these agents might allow for more effective chemotherapy. Whether the pro-apoptotic effects of low micromolar AKBA concentrations characterized here (Figures 1–6) have any clinical relevance, remains to be determined. The lack of specificity for the neoplastic phenotype of glioma cells (Figures 1 and 9) suggests that boswellic acids acquire clinical significance in neuro-oncology for their anti-oedema action, but not as tumour-cytotoxic agents. Yet, cytotoxicity studies with neonatal astrocytes, which are proliferating cells, as performed here, do not necessarily predict neurotoxicity in vivo, and preliminary studies in laboratory animals suggest that boswellic acids may inhibit the growth of glioma in the absence of intolerable side-effects (Winking et al, 1998).

### ACKNOWLEDGEMENTS

The authors thank Dr B Vogelstein (Baltimore, MD, USA) for p21$^{+/+}$, p21$^{+/-}$ and p21$^{-/-}$ human colon carcinoma cells, Dr A Strasser (Victoria, Australia) for the CRM-A expression plasmid, and Dr M Winking (Giessen, Germany) for valuable discussions. This study was supported by the BMBF and the Deutsche Forschungsgemeinschaft.

### REFERENCES

Ammon HPT (1996) Salai Guggal-Boswellia serrata: From a herbal medicine to a non redox inhibitor of leukotriene biosynthesis. *Eur J Med Res* **1**: 369–370

Böker DK and Winking M (1997) Die Rolle von Boswellia-Säuren in der Therapie maligner Gliome. *Deutsches Ärzteblatt* **94**: B958–B960

Gomez-Manzano C, Fueyo J, Kyritsis AP, McDonnell TJ, Steck PA, Levin VA and Yung WKA (1997) Characterization of p53 and p21 functional interactions in glioma cells en route to apoptosis. *J Natl Cancer Inst* **89**: 1036–1044

Gupta I, Parihar A, Malhotra P, Singh GB, Lüdtke R, Safayhi H and Ammon HPT (1997) Effects of boswellia serrata gum resin in patients with ulcerative colitis. *Eur J Med Res* **2**: 37–43

Heldt RM, Syrovets T, Winking M, Sailer ER, Safayhi H, Ammon HPT and Simmet Th (1997) Boswellic acids exhibit cytotoxic effects on brain tumor cells independent from 5-lipoxygenase inhibition. *Naunyn-S-Arch Pharmacol* **355**: 30 (Abstract)

Hoernlein RF, Orlikowsky Th, Niethammer D, Sailer E-R, Dannecker GE and Ammon HPT (1997) Acetyl-11-keto-β-boswellic acid (AKBA) induces apoptosis in HL60 and CCRF-CEM cells and inhibits topoisomerase I. *Soc Cancer Res* **1291** (Abstract)

Jung JM, Bruner JM, Ruan S, Langford LA, Kyritsis AP, Kobayashi T, Levin VA and Zhang W (1995) Increased levels of p21$^{WAF1/Cip1}$ in human brain tumors. *Oncogene* **11**: 2021–2028

 © Cancer Research Campaign 1999

Michalowitz D, Halevy O and Oren M (1990) Conditional inhibition of transformation and of cell proliferation by a temperature-sensitive mutant of p53. *Cell* **62**: 674–680.

Michieli P, Chedid M, Lin D, Pierce JH, Mercer WE and Givol D (1994) Induction of WAF1/CIP1 by a p53-independent pathway. *Cancer Res* **54**: 3391–3395

Naumann U, Durka S and Weller M (1998) Dexamethasone-mediated protection from drug toxicity linked to $p21^{WAF/CIP1}$ protein accumulation. *Oncogene* **17**: 1567–1575

Roth W, Fontana A, Trepel M, Dichgans J, Reed JC and Weller M (1997) Immunochemotherapy of malignant glioma: synergistic activity of CD95 ligand and chemotherapeutics. *Cancer Immunol Immunother* **44**: 55–63

Reber U, Wüllner U, Trepel M, Baumgart J, Seyfried J, Klockgether T, Dichgans J and Weller M (1998) Potentiation of treosulfan toxicity by the glutathione-depleting agent, buthionine sulfoximine, in human malignant glioma cells: the role of bcl-2. *Biochem Pharmacol* **55**: 349–359

Rowe TC, Chen GL, Hsiang YH and Liu LF (1986) DNA damage by antitumor acridines mediated by mammalian DNA topoisomerase II. *Cancer Res* **46**: 2021–2026

Ruan S, Okcu MF, Ren JP, Chiao P, Andreeff, Levin V and Zhang W (1998) Overexpressed WAF1/Cip1 renders glioblastoma cells resistant to chemotherapy agents 1,3-bis(2-chloroethyl)-1-nitrosourea and cisplatin. *Cancer Res* **58**: 1538–1543

Safayhi H, Mack T, Sabieraj J and Ammon HPT (1992) Boswellic acids: novel, specific non-redox inhibitors of 5-lipoxygenase. *J Pharmacol Exp Ther* **261**: 1143–1146

Safayhi H, Sailer ER and Ammon HPT (1995) Mechanism of 5-lipoxygenase inhibition by acetyl-11-keto-β-boswellic acids. *Mol Pharmacol* **47**: 1212–1216

Safayhi H, Rall B, Sailer ER and Ammon HPT (1997) Inhibition by boswellic acids of human leukocyte elastase. *J Pharmocol Exp Ther* **281**: 460–463

Sailer ER, Subramanian LR, Rall B, Hoernlein RF, Ammon HPT and Safayhi H (1996) Acetyl-11-keto-β-boswellic acid (AKBA): structure requirements for binding and 5-lipoxygenase inhibitory activity. *Br J Pharmacol* **117**: 615–618

Schulz JB, Weller M and Klockgether T (1996) A sequential requirement for new mRNA and protein synthesis, ICE-like protease activity, and free radicals in potassium deprivation-induced apoptosis of cerebellar granule cells. *J Neurosci* **16**: 4696–4706

Sheikh M, Chen YQ, Smith ML and Fornace AJ (1997) Role of $p21^{Waf1/Cip1/Sdi1}$ in cell death and DNA repair as studied using a tetracycline-inducible system in p53-deficient cells. *Oncogene* **14**: 1875–1882

Strasser A, Harris AW, Huang DC, Krammer PH and Cory S (1995) Bcl-2 and Fas/Apo-1 regulate distinct pathways to lymphocyte apoptosis. *EMBO J* **14**: 6136–6147

Trepel M, Groscurth P, Malipiero U, Gulbins E, Dichgans J and Weller M (1998) Chemosensitivity of human malignant glioma: modulation by p53 gene transfer. *J Neuro-Oncol* **39**: 19–32

Van Meir EG, Kikuchi T, Tada M, Li H, Diserens AC, Wojcik BE, Huang HJS, Friedmann T, De Tribolet N and Cavenee WK (1994) Analysis of the p53 gene and its expression in human glioblastoma cells. *Cancer Res* **54**: 649–652

Wagenknecht B, Gulbins E, Lang F, Dichgans J and Weller M (1997*a*) Lipoxygenase inhibitors block CD95 ligand-mediated apoptosis of human malignant glioma cells. *FEBS Lett* **409**: 17–23

Wagenknecht B, Trepel M, von Deimling A, Grimmel C, Rollbrocker B, Hayashi Y, Lang F, Dichgans J, Gulbins E and Weller M (1997*b*) p53 accumulation promotes dephosphorylation and proteolytic cleavage of retinoblastoma protein in human malignant glioma cells. *Cell Physiol Biochem* **7**: 304–311

Wagenknecht B, Schulz JB, Gulbins E and Weller M (1998) CD95L-induced apoptosis of malignant glioma cells: inhibition of the cascade by crm-A, bcl-2, and NDGA. *Cell Death Differ* **5**: 894–900

Waldman T, Kinzler KW and Vogelstein B (1995) p21 is necessary for the p53-mediated $G_1$ arrest in human cancer cells. *Cancer Res* **55**: 5187–5190

Waldman T, Lengauer C, Kinzler KW and Vogelstein B (1996) Uncoupling of S phase and mitosis induced by anticancer agents in cells lacking p21. *Nature* **381**: 713–716

Weller M, Frei K, Groscurth P, Krammer PH, Yonekawa Y and Fontana A (1994) Anti-Fas/APO-1 antibody-mediated apoptosis of cultured human malignant glioma cells. Induction and modulation of sensitivity by cytokines. *J Clin Invest* **94**: 954–964

Weller M, Malipiero UV, Aguzzi A, Reed JC and Fontana A (1995*a*) Protooncogene bcl-2 gene transfer abrogates Fas/APO-1 antibody-mediated apoptosis of human malignant glioma cells and confers resistance to chemotherapeutic drugs and therapeutic irradiation. *J Clin Invest* **95**: 2633–2643

Weller M, Malipiero U, Rensing-Ehl A, Barr P and Fontana A (1995*b*) Fas/APO-1 gene transfer for human malignant glioma. *Cancer Res* **55**: 2936–2944

Weller M, Schmidt C, Roth W and Dichgans J (1997*a*) Chemotherapy of human malignant glioma: prevention of efficacy by dexamethasone? *Neurology* **48**: 1704–1709

Weller M, Trepel M, Grimmel C, Schabet M, Bremen D, Krajewski S and Reed JC (1997*b*) Hypericin-induced apoptosis of human malignant glioma cells is light-dependent, independent of bcl-2 expression, and does not require wild-type p53. *Neurol Res* **19**: 459–470

Weller M, Winter S, Schmidt C, Esser P, Fontana A, Dichgans J and Groscurth P (1997*c*) Topoisomerase I inhibitors for human malignant glioma. Differential modulation of p53, p21, bax and bcl-2 expression and of CD95-mediated apoptosis by camptothecin and β-lapachone. *Int J Cancer* **73**: 707–714

Weller M, Rieger J, Grimmel C, Van Meir EG, De Tribolet N, Krajewski S, Reed JC, von Deimling A and Dichgans J (1998) Predicting chemoresistence in human malignant glioma cells: the role of molecular genetic analyses. *Int J Cancer* **79**: 640–644

Winking M, Sarikya S, Jödicke A and Böker DK (1998) Boswellic acids inhibit glioma growth. *J Cancer Res Clin Oncol* **124**: R141 (Abstr.)

Winter S, Roth W, Dichgans J and Weller M (1998) Synergy of CD95 ligand and teniposide: no role of cleavable complex formation and enhanced CD95 expression. *Eur J Pharmacol* **341**: 323–328

Winter S and Weller M (1998) Potentiation of CD95L-induced apoptosis of human malignant glioma cells by topotecan involves inhibition of RNA synthesis but not changes in CD95 or CD95L expression. *J Pharmacol Exp Ther* **286**: 1374–1382

© Cancer Research Campaign 1999
